# Vaccination Coverage and Adherence to Scheduling in Children Aged 0 to 18 Months: Effects of COVID-19 and Age

**DOI:** 10.3390/vaccines13040387

**Published:** 2025-04-03

**Authors:** Ubaldo Miranda-Soberón, Isabel Pino-Arana, Norma Pastor-Ramírez, Elena Figueroa-Cabezudo, Cyntia Zevallos-Parra, Gabriela Valencia-Borja

**Affiliations:** 1 Human Medicine Faculty, National University “San Luis Gonzaga”, Ica 11004, Peru; 2 Nursing Faculty, National University “San Luis Gonzaga”, Ica 11004, Peru; dpino@unica.edu.pe (I.P.-A.); npastor@unica.edu.pe (N.P.-R.); maria.figueroa@unica.edu.pe (E.F.-C.); 3 Seedbed CLIMA, Human Medicine Faculty, National University “San Luis Gonzaga”, Ica 11004, Peru; 20185431@unica.edu.pe (C.Z.-P.); 20183555@unica.edu.pe (G.V.-B.)

**Keywords:** child vaccination, vaccination coverage, vaccine adherence, Expanded Program on Immunization (EPI), COVID-19 effects

## Abstract

Vaccination in Peru began 50 years ago as part of the Expanded Program on Immunization (EPI), which has proven effective in saving the lives of millions of children. This research aimed to determine the coverage and adherence to the vaccination schedule in children up to 18 months of age during the period 2018–2022, including the COVID-19 pandemic lockdown, in order to assess its influence. Materials and methods: This was a secondary source study based on the Demographic and Family Health Survey (ENDES) of Peru, including a sample of 82,702 male and female children whose caregivers presented vaccination cards. Coverage and adherence indicators were calculated, and differences were evaluated between the pre-confinement, absolute confinement, and relative confinement periods using a chi-square test. Results: For almost all vaccines, coverage decreased from 2018 to 2022 (from 82.46% to 80.16% on average, *p* < 0.001). Coverage also decreased as the scheduled age increased (0–2 months: median 93%, 7–18 months: median 63%; *p* < 0.001). Average adherence rates also declined over time (2018: 65.82% to 2022: 61.77%). The most affected vaccine was the yellow fever vaccine. Coverage did not reach protective population levels, while adherence has averaged 85.06% since 2018. Conclusions: COVID-19 negatively influenced compliance with the vaccination schedule and adherence.

## 1. Introduction

Vaccines consist of antigens and adjuvants. Antigens are designed to stimulate an immune response, while adjuvants are substances that help to enhance and prolong the immune response [1]. Vaccines are useful for preventing disease and reducing complications, and have contributed to reducing child morbidity and mortality worldwide, leading to better child development [2].

The Expanded Program on Immunization (EPI) was established based on Resolution WHA 27.57, approved by the World Health Assembly in May 1974, with guidelines approved by the PAHO through Resolution CD25.R27 in 1977 [3]. In 1978, Peru established the National Health Services System (SNSS) through Decree Law 22365, which organized the health system more efficiently [4], including childhood vaccination. Since then, the vaccination schedule has evolved, adapting to technological advancements and disease control needs. Over time, vaccination rates have increased, and by 2015, they contributed to reducing child mortality in Peru [5]. However, maintaining these levels is challenging in times of rapid technological development, necessitating improvements and updates to vaccines and the vaccination system [6], with the particular aims of making the schedule accessible to caregivers [7] and delivering safer vaccines. A particular case was the crisis caused by the emergence of vaccine-derived polio cases in Peru [8].

Vaccines must be safe, effective, and available. To protect the population, their coverage must exceed 95% [9], and adherence to the vaccination schedule should be 100%. Adherence can be defined as “the process by which patients take their medications as prescribed” [10]. For vaccines, adherence begins with the first dose and continues until the schedule, including boosters, is completed. Delaying vaccination according to the schedule indicates a lack of adherence. Additionally, children do not make their own health decisions; these depend on their parents or caregivers and the conditions under which they make them [11,12]. It is widely accepted that vaccine refusal is complex, with arguments ranging from ethical, legal, and child rights issues to religious beliefs, distrust in the effectiveness of vaccines, potential harm, and others [13]. It is important to assess the magnitude of refusal and identify and characterize determinants, attitudes, and beliefs related to vaccination [14]. Negative attitudes are often influenced by anti-vaccine movements, which have been spreading worldwide [15]. Therefore, studying coverage and adherence is useful to determine whether the vaccination system is functioning properly or if changes are needed. However, depending on the data source and its handling, the results may be biased. The WHO recommends using surveys to obtain more accurate and valid estimates [16], especially when unforeseen events—such as the COVID-19 lockdown—may affect them.

In a study in China, Wen-yan Ji et al. [17] found that coverage for all vaccines except BCG and the first dose of hepatitis B decreased during the COVID-19 lockdown, and recommended flexible vaccination hours to avoid this. Bittencourt-Enorea et al. [18], in an ecological study in Brazil, observed a gradual decrease in coverage in some areas of Brazil and stationary trends in others, considering a 7-year period from 2015 to 2021. Butler AM et al. [19], using a secondary source in the United States, investigated whether there was a delay in the timing of children receiving their vaccines and found that, for each successive dose, more children did not receive their vaccine on time, demonstrating an impact on adherence. Michels SY et al. [20], studying children aged 0–24 months born between 2015 and 2017 in Montana, found that only 23.1% of Native American children received their vaccines on time, with delays at each scheduled age. Regarding coverage, at 24 months, only 56.6% of Native American children were vaccinated, a rate lower than that of non-Hispanic white children (64.3%) for all doses.

In this research, in order to determine vaccination coverage for the main vaccines scheduled in the first 18 months of life, analyze adherence levels to the vaccination schedule established in Peru, and assess the influence of the COVID-19 lockdown and the scheduled age, we analyzed the ENDES survey databases from the INEI ranging from 2018 to 2022, considering the following basic indicators: vaccination coverage, adherence levels to the schedule, and the child’s age when receiving scheduled vaccine doses.

## 2. Materials and Methods

### 2.1. Objective and Type of Research

The objective of this research was to determine the influence of the COVID-19 lockdown and the scheduled age on compliance with the vaccination schedule and adherence in children up to 18 months of age in Peru during the period 2018–2022. This is a secondary source, ecological study involving an annual temporal analysis.

### 2.2. Data

This research used the databases of the Demographic and Family Health Survey (ENDES) provided by the INEI of Peru [21], using only the modules related to child vaccination, namely, REC43 and REC95, for the years 2018, 2019, 2020, 2021, and 2022. It was not possible to use other modules as they are in separate databases and, in order to unite them, a common key variable is required, which they did not have, limiting the analysis of socio-demographic variables and other probable associated factors. This also limited the analysis by region.

In the ENDES survey, the unit of research was the usual residents of households. The target population was private households and their members, specifically, children under 5 years of age. The survey used balanced sampling (cube method), which allowed for better characterization of the population. Sampling was performed by clusters of households in the country’s regions. The survey was conducted from January to December each year. Regarding vaccines, the report only provided the percentage of children under 12–24 and 36 months with complete vaccines for their age.

For calculations, we analyzed the measurement years according to the categories of the Vaccination Card variable, as follows: does not have it, no longer has it, could not be observed, and could be observed. We only used the sample where the card was presented and could be observed and verified. We considered that the other categories constitute only the informant’s opinions, being based on their memory and therefore unverifiable. We also chose this category as only those who presented the card had the vaccination dates for the different vaccine doses, while those who did not show the card did not have them; this information was very important, as it was used to calculate the levels of adherence. The final sample was 82,702 children of both sexes (2018: 18,268, 2019: 17,422, 2020: 11,430, 2021: 17,956, and 2022: 17,626) (INEI. Demographic and Family Health Survey, ENDES) [21].

### 2.3. Variables and Indicators

The calculated indicators were as follows:

1. Vaccination coverage for each vaccine administered to male and female children in the first 18 months of life, by study year, according to vaccination age.
2. Adherence to the vaccination schedule for each vaccine administered to male and female children in the first 18 months of life, by study year, according to vaccination age.
3. The decimal age in months at which the child received each vaccine, in the evaluated years.

The vaccines analyzed, according to the vaccination card, were as follows: BCG0 (for tuberculosis, 1 dose at birth), HVB0 (against hepatitis B, 1 dose at birth), polio 2–4–6 (against polio, three doses at 2–4–6 months), pentavalent 2–4–6 (against diphtheria, pertussis, tetanus, hepatitis B, HIB; 3 doses at 2–4–6 months), pneumococcal 2–4–12 (against pneumococcus, 3 doses at 2–4–12 months), rotavirus 2–4 (against rotavirus, two doses at 2–4 months), influenza 7–8 (against influenza, two doses at 7–8 months), MMR 12–18 (against measles, mumps, and rubella, 2 doses at 12–18 months), and yellow fever 15 (against yellow fever, one dose at 15 months).

To assess the influence of the COVID-19 lockdown, we considered pre-confinement as 2018 and 2019; 2020 as absolute confinement, when the strictest restriction confinement was decreed and primary care facilities, which are the places where vaccines are usually applied to children, were closed, such that vaccination services were restricted (although the vaccination system was reactivated in the second half of that year) [22,23]; and 2021 and 2022 as relative confinement, when the vaccination system worked relatively normally [24].

With the birth and vaccination dates, we calculated the decimal age in months at which the child received each vaccine, allowing us to assess delays in vaccination and calculate adherence. For this, we set cut-off points and determined a window in which children were considered adherent, which was 15 days before the scheduled age to one month after, following the recommendations of the CDC [25] and other authors [20]. We accepted these cut-off points since the calendar is designed for completed age in months; we worked with decimal age. This is important due to the cultural, ethnic, geographic, and supply-related differences in accessibility across Peru’s different regions. Furthermore, we worked with time frames for vaccination, based on the required immune response. The program works with completed age in months.

### 2.4. Mathematical Analysis

With the data and calculated indicators, we constructed the following data: coverage graphs by year, box plots of coverage by scheduled age, cumulative percentage graphs of child age by cut-off points, graphs to identify adherence windows for protective and multiple vaccine doses, tables of coverage by vaccine and study year, and tables of adherence levels by year and vaccine type. Differences were measured using the chi-square test applied to the differences found in vaccination coverage by year and adherence levels by year for each vaccine and a Kruskal–Wallis test for analysis of difference in medians of coverage data grouped by scheduling age in the calendar. In addition, for both coverage and adherence, the levels of effect magnitude by year of study were calculated, using the OR with its confidence intervals and homogeneity test and linear trend, which allowed us to assess trends. The confidence intervals for coverage and adherence were also calculated (expressed as a percentage), considering *p* < 0.05 as significant. Mathematical calculations and graphing were performed using InfoStat/L free version 2017, LibreOffice 24.2.7, and EPIDAT v.3.

Information on factors associated with coverage levels could not be obtained, and the analysis was limited by the information available in the databases.

## 3. Results

A total of 82,702 children of both sexes were included in this research during the period 2018–2022, whose caregivers presented vaccination cards, demonstrating the vaccines received and the dates they were administered. Cases without a card, those that could not be observed, and those who lost it were excluded. Nine vaccines scheduled from birth to 18 months of age, according to Peru’s EPI, were investigated.

Regarding vaccination coverage, the highest rates were for vaccines administered in the first months of life; BCG0, administered at birth, reached a range of 92.71–95.87%; hepatitis B0 at birth was slightly lower (86.38 to 89.12%), with some years reaching 95%, considered a protective level. Vaccines administered at two months also reached high values, with some above 95%, the figure indicated to maintain a protected population.

Vaccines scheduled for three doses showed a gradual decrease in coverage from the first to the third dose, for all evaluated years, with averages of 93.53% for the first dose, 88.84% for the second dose, and 78.12% for the third dose (*p* < 0.05). The same occurred with two-dose vaccines. The yellow fever vaccine, administered at 15 months, had low coverage in all evaluated years (average 56.78%); the second dose of MMR18 at 18 months was even lower (average 52.02%). Variations in coverage were observed according to the evaluation years, both during the pre- and relative confinement stages, which were significant for all vaccines and scheduled doses (*p* < 0.001; see Table 1). This evidenced the influence of the lockdown during the pandemic, causing a decrease in coverage. Regarding the effect size, in the case of anti-polio6, a similar OR was obtained between 2018 and 2019 (without confinement); however, in 2020—the year of absolute confinement—the difference in unvaccinated people was 19.03% higher, while in the years of relative confinement, this diference 11.89% and 14.75% higher, which shows the influence of the level of confinement on vaccination coverage (*p* = 0.0001). All other vaccines presented similar results. Confidence levels varied little with respect to the calculated coverage.

**Figure 1:**
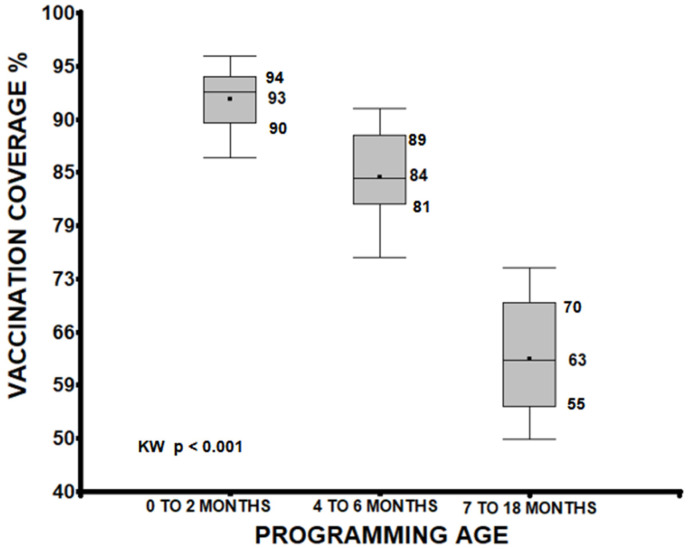
Age quartiles of the child when receiving the protective dose of the vaccine, considering only those who had a card with the vaccination date. There were also differences between the groups (*p* < 0.001).

A median analysis of vaccination coverage, grouping doses administered between 0 to 2 months, 4 to 6 months, and 7 to 18 months, identified significant differences between these groups. Vaccines from 0 to 2 months showed higher coverage (median 93%) than those from 4 to 6 months (median 84%) and 7 to 18 months (median 63%). A progressive decrease was observed as the scheduled age increased (*p* < 0.001), and the groups were also different from each other (0 to 2 vs. 4 to 6, 0 to 2 vs. 7 to 18, 4 to 6 vs. 7 to 18 months *p* < 0.001; see Figure 1).

**Figure 2:**
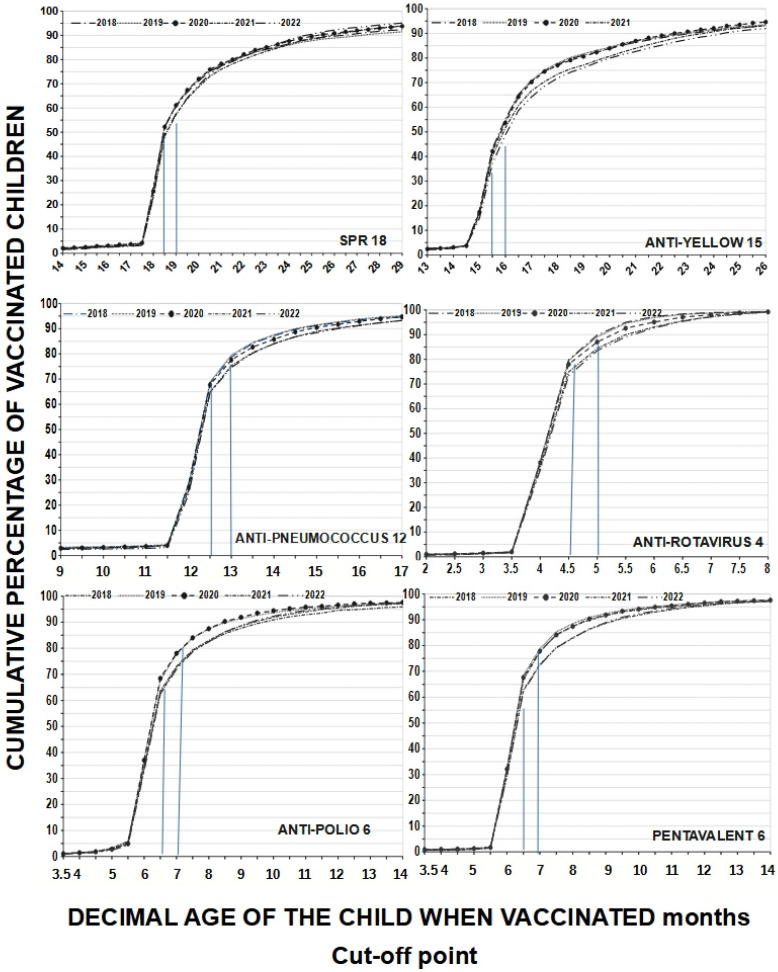
Adherence to the vaccination schedule according to cumulative percentages of vaccinated children by age at which they were vaccinated and type of vaccines received in protective doses by year evaluated (from 2018 to 2022); cut-off points are represented by blue lines. We see that adherence increases with increasing decimal age, but those vaccinated between 6.00 and 6.99 months are in the 6-month-old category.

A child may have received all vaccines but at the wrong time, reflecting a lack of adherence to the schedule. If only the coverage indicated on the vaccination card according to MINSA is considered, adherence at 18 months would present an average of 25.28% for MMR18, which is quite low; however, using the 18.5-month cut-off, adherence increases to 50.86% on average (difference 25.28%) and when accepting a one-month cut-off, it further increases to 59.56% (difference 8.7%). For yellow fever15, adherence at exactly 15 months was 16.74%, at 15.5 months it was 41.06%, and at the 15.99-month cut-off, it rose to 52.8%; as such, the observed differences were between 24.32% and 36.6%. Adherence at 6 months averaged 35.1% for polio6 but, at the 6.5-month cut-off, adherence rose to 65% (difference 29.9%), and at the 6.99-month cut-off, it further reached 74.64%. This increase in adherence is the result of working with decimal age. If we consider age in completed months, we would only have the total number of adherents for that age, which would be equal to the range of 6.00 to 6.99 months decimal age. These children are in the 6-month-old category, which is not very accurate. The behavior was similar considering the cut-off points for other vaccines. Assuming that different cut-off points provide different adherence levels, and in line with other authors, we chose cut-off points of 0.50 months before to one month after the scheduled age to analyze our coverage windows. On the other hand, this also changes the slopes of the curves, especially those for multiple-dose vaccines (see Figure 2).

Protective doses also matter for adherence, as it is assumed that the last scheduled dose of a vaccine provides adequate protection. This applies to vaccines with multiple doses, as single-dose vaccines generate immunity without needing additional doses. A child may receive all doses, but the highest immunity is achieved only if they are received at the prescribed times. Being adherent to the last dose is not the same as being adherent to all doses. Our data show that, for all multiple-dose vaccines, adherence decreases when evaluating the adherence to all doses compared to adherence to the last dose; for rotavirus4, 88.0% was achieved while, for total rotavirus 86.7% was achieved in 2018 and, in 2022, it was 81.9% vs. 80.2%, with a difference of less than 2%. The largest differences were observed for pneumococcal12 and total pneumococcal: pre-confinement was 75.0% vs. 66.35%, while relative confinement 71.4% vs. 59.15%. As such, the pre-confinement difference was 8.65%, while for relative confinement it was 12.25% (*p* < 0.001). Similar differences were observed for all multiple-dose vaccines. The lowest adherence was for yellow fever15: pre-confinement, 51.05% and relative confinement, 46.7% on average (*p* < 0.001). For MMR18, we observed pre-confinement, 56.9% and relative confinement, 54.1% on average (*p* < 0.001). For the last doses, adherence ranged from 85.06% to 49.14%; for multiple doses, it ranged from 83.54% to 63.34% (see Table 2). When we evaluated the level of effect of adherence to all doses of pentavalent, it was observed that the results for the years 2021 and 2022 (relative confinement) differed from those in the year 2018; in 2021, there were 18.85% more non-adherent people than in the year of comparison, which increased to 19.76% in 2022. All the vaccines evaluated a similar trend, except for the anti-polio6, which had a flat linear trend (*p* = 0.0636) and the total anti-polio also had a flat linear trend (*p* = 0.717). The confidence intervals were very close to the sample adherence values.

## 4. Discussion and Comments

This research was conducted to evaluate vaccination coverage and adherence to the vaccination schedule in children up to 18 months of age, considering the influence of the COVID-19 lockdown and vaccination age. Coverage below 95% was found for almost all vaccines in the five evaluated years (yearly averages: 2018, 82.31%; 2019, 82.46%; 2020, 79.82%; 2021, 79.85%; and 2022, 80.16%), with only the BCG, polio2, and pneumococcal2 vaccines in 2018 and 2019 presenting coverage above 95%. A significant association was found between coverage levels and the evaluated years, considering pre-confinement, absolute confinement, and relative confinement periods, with decreases in most cases (especially in 2021 and 2022). A noticeable decrease in vaccination coverage was observed as the child’s scheduled age increased (vaccines scheduled at 0–2 months: median 93%, 4–6 months: 84%, 7–18 months: 63%, KW, *p* < 0.001). The most affected vaccines were yellow fever15 and MMR18 (2018: 57.65% and 51.16%; 2019: 57.99% and 53.53%; 2020: 58.00% and 54.58%; 2021: 55.44% and 50.24%; and 2022: 54.84 and 50.58%, respectively), resulting in very low coverage. Vaccines that were applied in several doses also decreased in coverage as the number of doses increased and, in the end, population protective levels were not reached. Regarding adherence to vaccination, according to our cut-off points, except for anti-rotavirus4, which reached an average adherence of 85.06%, none of the others even reached 80%. The the averages per year for all vaccines were, from 2018 to 2022, 65.82%, 67.43%, 66.53%, 62.45%, and 61.77%, respectively. These averages cover all vaccines considered in this research. Adherence decreased in the years of relative confinement, compared to pre-confinement, for almost all vaccines (*p* < 0.01); the trend also decreased as the scheduled age increased (*p* < 0.01). The confidence intervals for both coverage and adherence were quite close to the sample value. The effect size levels also showed a greater number of unvaccinated or non-adherent vaccines as the years advanced (*p* < 0.01), except for anti-polio.

In recent studies, some researchers have reported immunization coverage in children that could be considered adequate; however, a tendency to decrease as the different vaccine doses are applied was also observed [26,27,28]. Shahid S et al. [29], in their research carried out in Pakistan, found that the highest coverage was for the pentavalent vaccine (72.8%), and the oral polio vaccine reached a coverage level of 69.2%; however, the measles vaccine was only 29.3%. They also found that coverage decreased with increasing vaccination age. Rauniyar S.K., et al. [30] reported coverage in Nepal ranging between 91.5% and 97.8% for different vaccines, which decreased with the age of vaccination. Other authors, such as Okello G, et al. [31] observed differences by geographic region. In our research, a relationship was found between the COVID-19 confinement period and vaccination coverage, in line with the findings of other authors. For example, Cooper S. et al. [32] reported an increase in vaccination coverage during the pandemic, from 50% at the beginning to 65% in 2021, (although, in one district, there was a significant drop from 65% to 32%). Regarding basic vaccines, a positive trend was observed before and during the pandemic, but was negative in other places: the range was from 59.0–69.0% to 57.0–83.0% in region A, and from 62.0–72.0% to 49.0–78.0% in region B. However, the author believed that this was only indicative of an increase in variability between regions. Albertsen N. et al. [33] reported a national coverage of 85.4% in Greenland, which varied from 79.65 to 91.7% in different localities. By vaccine, the variation ranged from 97.1% for TB to 64.1% for MMR, without having reached at least the 90% suggested by the WHO in the latter; the highest rates were obtained for vaccines administered at birth, while those administered at 15 months presented much lower rates. This is similar to what we found, rates below the WHO recommendations (e.g., 95% for measles).

Regarding the evaluation of adherence to the vaccination system, it is necessary to determine the time ranges in which it could be said that a child is adherent. Michels S.Y. et al. [20] considered up to one month after the age at which the dose was recommended, a criterion that we also used in this research (see graph no. 4). The same was considered by Newcomer S.R., et al. [34]. This measurement is important as a child can receive all their scheduled vaccine, but all or some of them may be received at the wrong time, failing to consider the vaccination schedule. In 2024, Butler A.M., et al. [19] reported that, at 2 months of age, up to 83% of children received the recommended vaccines on time, while at 4 months, this value was 72%, and that at 6 months, this was only 62%; notably, in 13% of children, no dose was timely. In children aged between 12 and 15 months, 6% had no timely vaccines, and there was more variation in adherence in the second compared to the first year of life. On the other hand, Rauniyar S.K., et al. [30] reported a general coverage of greater than 90% in Nepal; however, on-time vaccination varied from 41.5% for the pentavalent third dose to 73.9% for the pentavalent first dose. As for measles, only 53.8% were adherent and, for the anti-polio vaccine, the delay increased as the dose increased (first dose, 21.8%; second dose, 38.1%; third dose, 57.9%), similar to the results found in our research. In Alaska, Michels S.Y. et al. [20] found that at 24 months of age, 23.1% of Native American children had received all doses of seven combined vaccines on time, while 43.4% did not receive the vaccine or received them late. As such, disparity was found according to the ethnicity of the children (for DPT, 7 41.9% in Natives Americans vs. 70.4% in non-Native Americans). In the present research, higher adherence levels were observed; however, this could be due to the cut-off points.

Regarding the impact of COVID-19 on vaccination, in 2023, Novaes J.V. et al. [35], evaluating the period 2018–2021 in Brazil, found a decrease in coverage in the anti-pneumococcal vaccine, with a difference of 21.43% throughout the period. While they determined variations in adherence between states, overall, there was a 0.67% increase throughout the country. In the United States, Carias C. et al. [36] found an 8% decrease in measles vaccination; however, it recovered when the restrictions were lifted, increasing to 15% coverage. In China, Wen-yan Ji et al. [17] reported a decrease in adherence for the majority of regions. On the other hand, Bittencourt-Enorea et al. [18] reported a decrease in only some areas of Brazil. As can be seen from these studies, the majority of results indicated a decrease in coverage during confinement and in the following years, with figures higher than those found in this study. This would be explained by the actions undertaken by the Ministry of Health of Peru (MINSA), which, in 2020 decreed [22] measures to operationalize immunizations in the context of COVID-19, and, in July of the same year, approved the gap recovery plan, giving only three months for its execution; meaning that, in October, the coverage had recovered [23]. The results of this were measured by the ENDES 2020 pollsters; in Peru, this is called house-to-house sweeping for vaccination.

Considering the regional scope is important due to socio-economic similarities. Robini et al. [37] analyzed vaccination coverage, considering children under one year of age in Latin American countries in 2020, the year of greatest confinement in Peru and found some countries with coverage higher than 80%, such as Chile, Panama, and Nicaragua; the coverage in Peru was similar to that of Uruguay, Colombia, and Cuba, being above 60%. They concluded that the regional average scores in some countries fell in 2020, except for Chile and Colombia. Castrejona M. et al. [38] also carried out studies in Latin America, covering the years from 2017 to 2020. They observed an increase in the coverage of anti-rotavirus and pentavalent, and a decrease in BCG in Mexico; meanwhile, in Colombia, coverage in 2020 also decreased, even for BCG (between 1 and 6%). In Brazil, BCG coverage decreased by 5–12%, but those for the pentavalent and DPT vaccines increased. In Argentina, the downward trend in coverage was noticeable before the pandemic and continued in 2020; meanwhile, in Chile, coverage decreased by 3–6%, but that for BCG vaccines increased. They also analyzed Peru, observing a decrease in coverage of 6–16%, similar to what we found.

It is important to remember that having high coverage does not necessarily mean a protected population, delays in vaccination could disable such protection. Therefore, the child who goes to be vaccinated and does so in a timely manner is truly protected; and, therefore, we may have fewer protected children than we think. If we are strict in our calculations, and, in a population of 10,000 children we have 80% vaccinated with an adherence rate of 60%, we would have only 4800 children (or 48%) who are well protected. Of course, even if only one dose of vaccine is administered, the child acquires a certain level of protection. Therefore, it is necessary to continue evaluating vaccination systems by analyzing coverage, to which we must add adherence and, better yet, evaluation of the levels of protective antibodies in the blood [39]. Only in this way can we obtain a clear picture of the real efficiency of our vaccination system for children. In addition, it is necessary to investigate the safety of vaccines and the safety of the vaccination schedule.

In the present case, we have intermediate-level vaccination coverage, which should be increased to the levels recommended by international organizations for each vaccine (generally greater than 95%), as this allows for the protection of people for whom vaccination is contraindicated, such as those with allergies or immunodeficiencies [40], or those who do not wish to be vaccinated for religious or other reasons [41]. In children, this is a bigger problem; as they cannot decide on their health care, the decision is being made by their caregivers [42]. Not only do caregivers decide whether to take them to be vaccinated, but also when to do so; hence, education aimed at these adults is critically important [43]. It is also necessary to include the participation of children in these decisions, when they are willing and able to do so [44]. There are many strategies in this regard, described in the different manuals and research reports [45] for each vaccine. It is unquestionable that all health professionals must be trained for this task, especially nurses, who are directly related to the act of vaccination and have much more opportunity to do so, but they must be motivated and trained [46]. Promotion campaigns can give positive results in order to increase coverage [47], and we must not forget the current role of social networks on the Internet, which are an excellent social communication system that should be used [48]. Furthermore, the strategy of ”sweeping” house to house with mobile vaccination teams is useful, although potentially expensive [49].

Regarding the strategies to improve vaccination coverage, in addition to those mentioned in the previous paragraph, others have been studied by Oyo-Ita A. et al. [50], who published a meta-analysis on the interventions designed to improve immunization coverage in children living in middle-income countries, finding that health education and household records can improve coverage, while telephone calls and reminders do not adequately improve vaccine acceptance. However, the intervention of community leaders associated with the provider can increase acceptance. Complete vaccination of children under 2 years of age can be improved through training health workers in communication skills with people and integrating vaccination with other health activities. In countries in the Americas, among others, these combined strategies could be used, in addition to those already applied, to optimize coverage.

Among the limitations of this research, we have noted that errors were found in terms of filling out the vaccination date, which prevented the vaccination age for some vaccines from being calculated. Coding and transcription errors are often present, as are cases lost from the system; however, a sufficient sample was obtained. On the other hand, all cases that were based on the caregiver’s memory and had no tangible verification with respect to the vaccination card were excluded. Spatial analysis by region was not possible, which could be the subject of further research. Causal relationships by subject were not calculated, which could provide relevant information, as this study followed a global ecological design, aimed at investigating general trends that should be used in decision-making to propose health policies. Only coverage and adherence of vaccines applied in the first 18 months of life were calculated. Other factors that could be associated with vaccination coverage were not analyzed either. The data sources used were those of the ENDES survey, which is conducted annually in Peru. These are cross-sectional and possess the usual errors of this type of study, although their usefulness is indisputable. In addition, after the survey, it is likely that parents reflected on the usefulness of the vaccines and went to complete their children’s schedules, which could have modified the real results; however, this effect was not considered in this research.

## 5. Conclusions

- Vaccination coverage decreased from the first to third dose in multiple-dose vaccines.
- In general, vaccination coverage was low (less than 95%), leading to poor protection of the population.
- The older the scheduled age, the lower the coverage.
- The lockdown due to the COVID-19 pandemic reduced coverage for all vaccines.
- The lockdown due to the pandemic affected adherence to the vaccination schedule, which was consistent despite the use of different cut-off points.
- Adherence decreases as vaccination age increases.

## Figures and Tables

**Table 1:** Vaccination coverage by type of vaccine, dose and year of evaluation.

Vaccine Administered According to Schedule	Vaccination Coverage Year of Evaluation										
	2018 Total: 18,268		2019 Total: 17,422		2020 Total: 11,430		2021 Total: 17,956		2022 Total: 17,626		
	N°	%	N°	%	N°	%	N°	%	N°	%	X^2^ *p*
BCG0	17,440	95.47	16,702	95.87	10,617	92.89	16,647	92.71	16,514	93.69	0.001
ANTI-HEPATITIS0	15,806	86.52	15,323	87.95	9884	86.47	15,659	87.21	15,708	89.12	0.001
PENTAVALENT2	17,248	94.42	16,546	94.97	10,399	90.98	16,584	92.36	16,442	93.28	0.001
PENTAVALENT4	16,382	89.68	15,704	90.14	9823	85.94	15,868	88.37	15,620	88.62	0.001
PENTAVALENT6	15,253	83.50	14,568	83.62	9160	80.14	14,649	81.58	14,411	81.76	0.001
ANTI-POLIO2	17,539	96.01	16,753	96.16	10,570	92.48	16,805	93.59	16,420	93.16	0.001
ANTI-POLIO4	16,598	90.86	15,898	91.25	9988	87.38	16,044	89.35	15,740	89.30	0.001
ANTI-POLIO6	15,412	84.37	14,734	84.57	9303	81.39	14,815	82.51	14,464	82.06	0.001
ANTI-PNEUMOCOCCUS2	17,515	95.88	16,696	95.83	10,327	90.35	16,444	91.58	16,288	92.41	0.001
ANTI-PNEUMOCOCCUS4	16,576	90.74	15,791	90.64	9772	85.49	15,737	87.64	15,456	87.69	0.001
ANTI-PNEUMOCOCCUS12	13,098	71.70	12,220	70.14	7923	69.32	12,154	67.69	11,963	67.87	0.001
ANTI-ROTAVIRUS2	17,087	93.54	16,316	93.65	10,103	88.39	15,956	88.86	15,825	89.78	0.001
ANTI-ROTAVIRUS4	15,405	84.33	14,870	85.35	9227	80.73	14,561	81.09	14,349	81.41	0.001
ANTI-INFLUENZA6	14,196	77.71	13,248	76.04	8677	75.91	13,568	75.56	13,323	75.59	0.001
ANTI-INFLUENZA7	12,011	65.75	10,897	62.55	7225	63.21	10,906	60.74	10,780	61.16	0.001
ANTI-YELLOW15	10,549	57.75	10,103	57.99	6629	58.00	9955	55.44	9666	54.84	0.001
SPR12	13,608	74.49	12,885	73.96	8364	73.18	12,702	70.74	12,430	70.52	0.001
SPR18	9361	51.24	9326	53.53	6238	54.58	9021	50.24	8915	50.58	0.001

Of interest are the coverage rates for all vaccines in the protective doses. First, only the BCG vaccine reached high and protective levels (>95%) in the pre-confinement years (95.67%), which significantly decreased during absolute confinement and relative confinement (95.67% vs. 93.10% relative confinement, *p* < 0.01). None of the other protective doses reached adequate coverage in the three periods evaluated; rather they decreased in relative confinement. They decreased even more for yellow fever15 (56.80% on average) and MMR18 (52.03% on average).

**Table 2:** Adherence to vaccination schedule by year investigated and vaccine dose that produces the best protection.

		Year of Evaluation										
	Adherence	2018		2019		2020		2021		2022		X^2^ *p*
Protective Vaccine		N°	%	N°	%	N°	%	N°	%	N°	%	
ANTI-ROTAVIRUS4	YES	11,686	88.0	11,435	87.5	7009	85.2	10,768	82.7	10,569	81.9	0.000001
	NOT	1594	12.0	1634	12.5	1214	14.8	2253	17.3	2341	18.1	
	TOTAL	13,280		13,069		8223		13,021		12,910		
ANTI-POLIO6	YES	9804	67.0	10,181	72.3	6551	73.3	9677	68.3	9315	67.8	0.00001
	NOT	4822	33.0	3898	27.7	2391	26.7	4497	31.7	4428	32.2	
	TOTAL	14,626		14,079		8942		14,174		13,743		
PENTAVALENT6	YES	9977	76.1	9840	77.2	6106	76.4	9110	71.0	9022	70.9	0.000001
	NOT	3136	23.9	2899	22.8	1890	23.6	3723	29.0	3708	29.1	
	TOTAL	13,113		12,739		7996		12,833		12,730		
ANTI-NEUMOCOCUS12	YES	8344	74.8	7998	75.2	5174	73.6	7732	71.4	7631	71.4	0.00002
	NOT	2818	25.2	2638	24.8	1852	26.4	3099	28.6	3056	28.6	
	TOTAL	11,162		10,363		7026		10,831		10,687		
ANTI-YELLOW15	YES	4631	51.5	4433	50.6	2854	50.20	4125	48.00	3790	45.40	0.0002
	NOT	4365	48.5	4321	49.4	2836	49.80	4474	52.00	4563	54.60	
	TOTAL	8996		8754		5690		8599		8353		
SPR18	YES	4541	56.9	4589	56.9	3128	57.00	4318	53.60	4349	54.60	0.00002
	NOT	3435	43.1	3476	43.1	2357	43.00	3738	46.40	3614	45.40	
	TOTAL	7976		8065		5485		8056		7963		
FULL ANTI-ROTAVIRUS	YES	11,507	86.7	11,264	86.2	6850	83.3	10,593	81.3	10,357	80.2	0.000001
	NOT	1773	13.3	1805	13.8	1373	16.7	2428	18.7	2553	19.8	
	TOTAL	13,280		13,069		8223		13,021		12,910		
FULL ANTI-POLIO	YES	9060	61.9	9613	68.3	6202	69.4	9114	64.4	8726	63.5	0.00001
	NOT	5566	38.1	4466	31.7	2740	30.6	5040	35.6	5017	36.5	
	TOTAL	14,626		14,079		8942		14,154		13,743		
FULL PENTAVALENT	YES	9595	73.2	9502	74.6	5889	73.6	8741	68.1	8640	67.9	0.00001
	NOT	3518	26.8	3237	25.4	2107	26.4	4092	31.9	4090	32.1	
	TOTAL	13,113		12,739		7996		12,833		12,730		
FULL ANTI-NEUMOCOCCUS	YES	7223	64.7	7048	68.0	4617	65.7	6423	59.3	6306	59.0	0.00001
	NOT	3939	35.3	3315	32.0	2409	34.3	4408	40.7	4381	41.0	
	TOTAL	11,162		10,363		7026		10,831		10,687		

## Data Availability

All of this is considered in materials and methods, the reference is the number 21 INEI. Encuesta Demográfica y de Salud Familiar ENDES. Microdatos. [Internet]. 2024. Available online: https://proyectos.inei.gob.pe/microdatos/ (accessed on 15 October 2024).
